# Synthesis and Antioxidant Activity of Polyhydroxylated *trans*-Restricted 2-Arylcinnamic Acids

**DOI:** 10.3390/molecules20022555

**Published:** 2015-02-02

**Authors:** Mitko Miliovsky, Ivan Svinyarov, Elena Prokopova, Daniela Batovska, Simeon Stoyanov, Milen G. Bogdanov

**Affiliations:** 1 Faculty of Chemistry and Pharmacy, University of Sofia “St. Kliment. Ohridski”, 1, James Bourchier Blvd., Sofia 1164, Bulgaria; E-Mails: lishpera_87@abv.bg (M.M.); ivansvinyarov@abv.bg (I.S.); 2 Institute of Organic Chemistry with Centre of Phytochemistry, Bulgarian Academy of Science, Acad. G. Bonchev Str. Bl. 9, Sofia 1113, Bulgaria; E-Mails: elenatodorova90@gmail.com (E.P.); danibat@orgchm.bas.bg (D.B.); s_stoyanov@orgchm.bas.bg (S.S.)

**Keywords:** homophthalic anhydride, ROS, antioxidants, phenolic acids, hybrid molecules, synergy

## Abstract

A series of sixteen polyhydroxylated *trans*-restricted 2-arylcinnamic acid analogues **3a**–**p** were synthesized through a one-pot reaction between homophthalic anhydrides and various aromatic aldehydes, followed by treatment with BBr_3_. The structure of the newly synthesized compounds was confirmed by spectroscopic methods and the configuration around the double bond was unequivocally estimated by means of gated decoupling ^13^C-NMR spectra. It was shown that the *trans*-cinnamic acid fragment incorporated into the target compounds’ structure ensures the *cis*-configuration of the stilbene backbone and prevents further isomerization along the carbon–carbon double bond. The antioxidant activity of compounds **3a**–**p** was measured against 1,1-diphenyl-2-picrylhydrazyl (DPPH^●^), hydroxyl (OH^●^) and superoxide (O_2_^●▬^) radicals. The results obtained showed that the tested compounds possess higher activities than natural antioxidants such as protocatechuic acid, caffeic acid and gallic acid. Moreover, it was shown that a combination of two different and independently acting fragments of well-known pharmacological profiles into one covalently bonded hybrid molecule evoke a synergistic effect resulting in higher than expected activity. To rationalize the apparent antioxidant activity and to establish the mechanism of action, a SAR analysis and DFT quantum chemical computations were also performed.

## 1. Introduction

**Figure 1:**
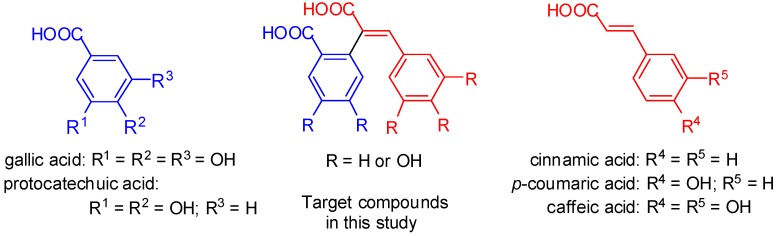
Structure of natural phenolic acids targeted as active fragments for the synthesis of hybrid antioxidants.

Various Reactive Oxygen Species (ROS) such as superoxide radical anion (O_2_^●▬^), hydroxyl radical (HO^●^) and hydrogen peroxide (H_2_O_2_) are essential cellular components, enzymatically generated in aerobic living organisms, which play an important role in different physiological and pathological processes. Particularly, at low levels, ROS take part in signal transduction, gene transcription and regulation of soluble guanylate cyclase activity [1,2,3]. In contrast, the accumulation of excessive ROS, mainly due to external influences such as radiation, ultraviolet light, cigarette smoke, pathogens, drugs, *etc*., can inflict damage upon cellular macromolecules—DNA, proteins and lipids—thus contributing to the development of various diseases, including diabetes mellitus, atherosclerosis, myocardial infarction, ischemia, epilepsy, anemia and carcinogenesis, to name just a few [4,5,6,7]. Healthy organisms are able to maintain the balance between the available reactive species and the antioxidant systems, but in some pathological circumstances, the endogenous antioxidants are not enough to deal with the increased levels of ROS [8,9]. Thus, in order for the level of excessive ROS to be reduced, and so the oxidative damage can be suppressed, the need for additional intake of exogenous antioxidants can be suggested [10,11]. Indeed, various natural and synthetic phenolic antioxidants such as stilbenoids, coumarins, flavonoids and carboxylic acids are part of our everyday diet, and it is nowadays truly believed that they are responsible for the prevention of many diseases associated with the oxidative stress [12,13,14,15,16,17,18,19]. However, the beneficial effect of these antioxidants seems to be conditioned by their limited distribution throughout the body, mainly due to their low lipophilicity [20,21]. To overcome this, experiments aimed at the synthesis of new antioxidants with improved properties from a pharmacological standpoint have been performed. Particularly, Borges and co-workers showed that the antioxidant properties of natural phenolic acids can be improved by introduction of functionalities which increase their lipophilicity [22,23,24,25,26,27,28,29]. Moreover, they have shown [30] that hybrid molecules consisting of gallic and cinnamic acid fragments (see Figure 1) possess significantly improved absorption, distribution, metabolism, and excretion (ADME) properties, which suggests their potential as drugs for the treatment of oxidative stress associated diseases. 

Recently we reported a convenient approach for the synthesis of polyhydroxy *cis*-restricted stilbenes, structurally based on two independent fragments such as protocatechuic acid and cinnamic acid (Figure 1), and demonstrated that the synthesized compounds possess a triple biological action as potent radical scavengers, antifungal agents and tyrosinase inhibitors at micromolar concentrations [31]. Furthermore, we showed that a combination of two different and independently acting fragments of well-known pharmacological profiles into one covalently bonded hybrid molecule evokes a synergistic effect resulting in higher than expected activity.

In continuation of this preliminary study, herein we present the synthesis of a more complete series of hybrid derivatives composed of natural phenolic acids. Their antioxidant capacity was evaluated by means of *in vitro* screening tests against 1,1-diphenyl-2-picrylhydrazyl (DPPH^●^), hydroxyl (OH^●^) and superoxide (O_2_^●▬^) radicals. The results obtained showed that most of the tested compounds are highly effective in micromolar concentrations, possessing higher activities than the conventional reference antioxidants. To rationalize the apparent antioxidant activity and to establish the mechanism of action, DFT quantum chemical computations and SAR analysis, which take into account the number and position of the hydroxyl groups in the carbon skeleton were also performed.

## 2. Results and Discussion

### 2.1. Chemistry 

**Scheme 1:**
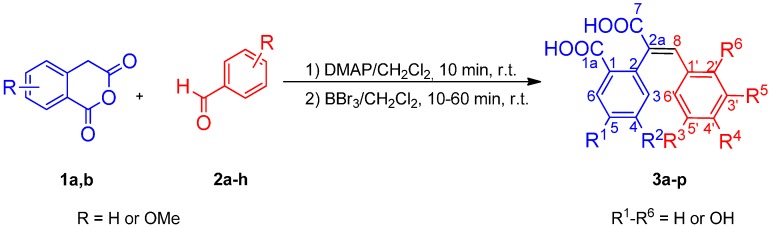
One-pot synthesis of polyhydroxylated *trans*-restricted 2-arylcinnamic acid. Numbering in the general formula **3** is used for NMR spectra description.

The compounds studied herein were obtained by means of a one-pot procedure for the synthesis of polyhydroxylated *cis*-restricted stilbenes recently published by us [31]. In brief (see Scheme 1), the method used consists of sequential reactions between homophthalic anhydrides **1** and aromatic aldehydes **2** to produce diastereomeric mixtures of the corresponding methoxylated 3-aryl-3,4-dihydro-isocoumarin-4-carboxylic acids [32,33], which, after treatment with BBr_3_, give the target polyhydroxylated compounds **3a**–**p** [31]. This way, a series of hybrid homologues consisting of benzoic, protocatechuic, cinnamic, caffeic, *p*-coumarinc, or gallic acids fragments were obtained in short reaction times (10–60 min). 

**Table 1:** Substitution patterns, number of hydroxyl groups, type of phenolic fragments, and antioxidant activity (EC_50_± SD) of compounds **3a**–**p** and standard antioxidants.

Comp.	R^1^	R^2^	R^3^	R^4^	R^5^	R^6^	Total OH	Type of Phenolic Fragments in Rings A/B	Antioxidant Activity ^a^			
									DPPH^●^		O_2_^●▬^ (µM)	HO^●^ (µM)
									EC_50_ (µM)	TEC_50_^b^ (min)		
**3a**	H	H	H	H	H	H	0	–/–	na ^c^	nd ^c^	na	201.0 ± 8.0
**3b**	H	H	H	H	H	OH	1	–/phenol	na	nd	na	133.7 ± 16.1
**3c**	H	H	H	OH	H	H	1	–/phenol	na	nd	na	134.2 ± 5.6
**3d**	H	H	H	OH	H	OH	2	–/resorcinol	41.30 ± 0.35	32.25 ± 2	na	120.9 ± 9.8
**3e**	H	H	OH	H	H	OH	2	–/hydroQ ^c^	6.74 ± 0.30	3.5 ± 0.1	na	122.9 ± 2.0
**3f**	H	H	H	H	OH	OH	2	–/catechol	6.26 ± 0.58	4 ± 0.1	372.9 ± 8.5	108.9 ± 14.0
**3g**	H	H	H	OH	OH	H	2	–/catechol	5.81 ± 0.29	2.75 ± 0.1	109.7 ± 5.3	59.4 ± 0.4
**3h**	H	H	OH	OH	OH	H	3	–/pyrogallol	4.47 ± 0.07	3 ± 0.1	11.1 ± 0.1	100.6 ± 2.1
**3i**	OH	OH	H	H	H	H	2	catechol/–	7.62 ± 0.28	31.75 ± 1.5	233.6 ± 0.9	118.7 ± 2.6
**3j**	OH	OH	H	H	H	OH	3	catechol/phenol	8.04 ± 0.32	26 ± 0.75	238.0 ± 5.4	79.1 ± 0.6
**3k**	OH	OH	H	OH	H	H	3	catechol/phenol	7.34 ± 0.25	33 ± 2	230.7 ± 4.9	64.5 ± 0.8
**3l**	OH	OH	H	OH	H	OH	4	catechol/resorcinol	5.43 ± 0.36	26.5 ± 0.75	269.2 ± 12.8	68.9 ± 0.8
**3m**	OH	OH	OH	H	H	OH	4	catechol/hydroQ ^c^	3.33 ± 0.04	10 ± 0.5	nd	58.8 ± 1.1
**3n**	OH	OH	H	H	OH	OH	4	catechol/catechol	3.52 ± 0.05	13 ± 0.5	137.2 ± 3.9	84.6 ± 2.3
**3o**	OH	OH	H	OH	OH	H	4	catechol/catechol	2.76 ± 0.12	6.5 ± 0.25	64.9 ± 1.9	42.6 ± 1.8
**3p**	OH	OH	OH	OH	OH	H	5	catechol/pyrogallol	2.09 ± 0.11	9 ± 0.75	11.3 ± 0.5	58.6 ± 0.9
Trolox	–	–	–	–	–	–	1	phenol	9.34 ± 0.07	5.75 ± 0.25	na	109.6 ± 7.8
**CA**	–	–	H	OH	OH	H	2	catechol	9.48 ± 0.17	9 ± 0.75	126 ± 10.6	73.0 ± 1.8
**PCA**	OH	OH	–	–	–	–	2	catechol	8.85 ± 0.24	28.25 ± 1.25	233.5 ± 3.0	117.7 ± 1.8
**GA**	–	–	–	–	–	–	3	pyrogallol	5.32 ± 0.34	10 ± 0.5	29.1 ± 1.0	146.9 ± 4.0
CA:PCA	–	–	–	–	–	–	2	catechol + catechol	13.28 ± 0.54	10 ± 0.5	143.9 ± 5.7	135.7 ± 13.1

^a^ Results are presented as a mean value of three measurements and the corresponding *p*-values of all possible pairs of compounds are given as supporting information (Tables S1–S3); ^b^ TEC_50_—the time required for each compound to reach equilibrium at concentration equal to EC_50_; ^c^ “na” refers to “not active”, “nd” refers to “not determined”, “–” refers to “not applicable”, and “hydroQ” refers to hydroquinone.

All compounds **3a**–**p** (whose substitution patterns are given in Table 1) were isolated in pure form after flash-chromatography purification and their structures were elucidated by means of spectral methods (see Section 3.2). In the case of ^1^H-NMR, ^13^C-NMR and IR methods, the spectra were taken for the synthesized compounds, while their mass spectra were obtained after derivatization with ethereal diazomethane solution prior to analysis. It is noteworthy that when *ortho*-methoxy-benzaldehydes were used as starting materials, the compounds of type **3** were obtained in low yields (*ca.* 14%–32%) due to their tendency to undergo a subsequent rearrangement leading to coumarins as main products [34]. Nevertheless, the fast precipitation of the residue after the purification step resulted in the isolation of these compounds in pure form, which allowed us to assess the influence of the *ortho*-phenolic group in the cinnamic acid fragment on the antioxidant activity. In order to elucidate the configuration of the herein synthesized hybrid molecules an approach based on the heteronuclear coupling constants was applied, since it is known that the vicinal heteronuclear coupling constants exhibit a Karplus type relationship between their value and the dihedral angle between the interacting nuclei [35]. This approach has already proved successful and accurate for configurational studies of similar compounds, even when the diastereomers with the opposite configuration are not available [31,33]. The results obtained proved that the incorporated *trans*-cinnamic acid fragment into the target compounds structure ensures *cis*-configuration of the stilbene backbone and prevents further isomerization along the carbon–carbon double bond. For more detailed discussion on the configuration and conformation of compounds **3a**–**p**, see Section 2.3. 

### 2.2. In Vitro Antioxidant Capacity Assays 

The antioxidant capacity of the hybrid molecules **3a**–**p** was measured against 1,1-diphenyl-2-picrylhydrazyl (DPPH^●^), hydroxyl (HO^●^) and superoxide (O_2_^●▬^) radicals. The analyses were performed as described in the Experimental Section and the activities were expressed as EC_50_± SD (µM) values. The averages were statistically compared and this was taken into account throughout the following discussion. A level of confidence higher than 95% (*p* < 0.05) was considered as statistically significant. The individual *p*-values of each pair of compounds are given as supporting information (Tables S1–S3). For a comparison purpose, well-known antioxidants such as Trolox, protocatechuic acid (PCA), caffeic acid (CA) and gallic acid (GA) were used as reference compounds. The results obtained are presented in Table 1, and for the sake of simplicity, the aromatic rings of the benzoic acid and cinnamic acid fragments are respectively named as ring A and ring B.

#### 2.2.1. DPPH*^●^* Radical Scavenging Assay

The DPPH^●^-scavenging assay has been successfully applied to evaluate the activity of diverse natural and synthetic antioxidants [36,37,38,39], it is easy to perform, highly reproducible and therefore, this method was initially employed to assess the radical scavenging activity of the hybrid molecules synthesized by us. The following observed order: **3d** < CA ≈ Trolox < PCA < **3j** < **3i** ≈ **3k** < **3e** ≈ **3f** < **3g** < **3l** ≈ GA < **3h** < **3n** < **3m** < **3o** < **3p** suggests, that except for **3a**–**c** which showed no activity, the group of compounds tested demonstrated comparable or even higher radical scavenging activity than the standards used. As it can be seen from Table 1, **3a**–**c**, which possess no or a single OH group on ring B, are not able to scavenge DPPH radicals, the latter confirming the importance of the number of the OH groups for the antioxidant activity. Thus, despite it is the least active compound in the series, compound **3d** showed an EC_50_ = 41.3, and this can be attributed to the presence of only two *meta*-orientated OH groups (a resorcinol-like fragment) in ring B. Compounds **3e**–**g**, each of them possessing two OH groups capable of forming *ortho*- or *para*-quinone structures in the same fragment, showed slightly higher activity (EC_50_ = 5.81–6.74) than Trolox (EC_50_ = 9.34), PCA (EC_50_ = 8.85) and CA (EC_50_ = 9.48). Similarly, **3i**–**k**, possessing two or three OH groups, showed EC_50_ = 7.34–8.04, and this can be attributed to the presence of the same catechol moiety in ring A, which seems to be responsible for the apparent activity. Compound **3h** contains a pyrogallol fragment (three neighboring OH groups) and **3l** combines cathechol and resorcinol fragments (2 × 2 OH groups), which suggests an increased radical scavenging ability. Indeed, their EC_50_ values (4.47 and 5.43 for **3h** and **3l**, respectively) were found close to that of GA (EC_50_ = 5.32), and thus, the distribution of the OH groups in the basic carbon backbone can be considered of an immense importance in modulating the radical scavenging activity. Considering compounds **3a**–**h**, which differ only by the number of OH groups present in ring B, it can be seen that the presence of a single OH group either at the 2 or 4 position in ring B (**3b** and **3c**, respectively) did not affect the activity of **3a**, but the addition of a second or a third OH group did do so, significantly in some cases, and thus compounds **3e**–**h** possess one order of magnitude lower EC_50_ values compared to **3d**. Unlike compounds **3е**–**3l**, which have only one structural fragment capable of scavenging free radicals effectively, **3m**–**p** possess another one in addition to the catechol fragment in ring A. This way, they are the most active compounds in the series with EC_50_ = 3.33, 3.52, 2.76 and 2.09, respectively. The latter suggests a synergistic effect resulting from the combination of two active fragments in one hybrid molecule, which can be rationalized by a comparison of the activity of **3o** to that of CA, PCA and their equimolar mixture. As it can be seen, the whole is better than the parts, since the hybrid molecule possesses a three- to fourfold lower EC_50_ value due to its facilitated hydrogen transfer and better radical stabilization. 

#### 2.2.2. O_2_^●▬^ Radical Anion Scavenging Activity

Superoxide anion radical is one of the most dangerous ROS. It is the first free radical generated after oxygen uptake in living cells, and it can undergo further enzyme-catalyzed transformations into other harmful ROS such as hydrogen peroxide and hydroxyl radical [40]. In the present study, the radical scavenging activity of compounds **3a**–**p** toward O_2_^●▬^ was estimated by means of a method proposed by Nishikimi *et al*. [41], but with slight modifications. According to this method, the superoxide anion radical, formed from an oxygen molecule by the phenazine methosulfate (PMS)/nicotinamide adenine dinucleotide (NADH) system, reduces nitroblue tetrazolium (NBT) to a blue colored formazan, which can be quantified spectrophotometrically at 560 nm. A short retrospection of the results presented in Table 1, indicates that the ability of a particular molecule to scavenge O_2_^●▬^ is simultaneously dependent on the number and distribution of the OH groups into the basic carbon backbone. As it can be seen, Trolox and compounds **3a**–**e**, possessing no, one or two OH groups in ring B, show no activity. The latter suggests that the presence of “isolated” OH groups, regardless their number, is not enough to evoke activity and this can be rationalized by consideration of the results for compounds **3d**–**g**. Although each of them possesses two OH groups in ring B, **3d** and **3e** are not active, whereas **3f** and **3g** exhibit EC_50_ = 373.0 and 109.7, respectively. Consequently, the presence of a catechol fragment can be considered necessary for superoxide anion radical scavenging activity. Moreover, it seems that the position of the catechol fragment is also important, since **3g** and CA (possessing similar substitution patterns in ring B), are nearly fourfold more active than **3f**. Further, PCA and compounds **3i**–**l**, each of them possessing the same catechol moiety into ring A, showed similar EC_50_ values and so the increasing number of “isolated” OH groups in the cinnamic part again does not alter the apparent activity, until an additional catechol fragment does not appear in ring B. This way, the combination of two active fragments in **3n** and **3o** leads to a significant activity increase in comparison with that demonstrated by the individual parts or their equimolar mixture. Another interesting result deserves additional attention, namely, that the highest activity was demonstrated by compounds **3h** and **3p**, both of them containing pyrogallol fragments in ring B. As it can be seen, the two compounds show similar to each other and threefold lower EC_50_ values than that demonstrated by the strongest standard antioxidant—gallic acid—and this can be attributed to the presence of an extended π-conjugated system providing better delocalization. The latter also suggests that in case of several fragments capable of scavenging free radicals, the apparent activity can be dominated by those fragments which better stabilize the radicals formed. In sum, the catechol and pyrogallol fragments, as well as an extended π-conjugated system, can be considered as fragments of an immense importance when one intends to synthesize effective antioxidants capable of scavenging superoxide anion radicals. 

#### 2.2.3. HO^●^ Radical Scavenging Activity

Hydroxyl radical (HO^●^) can be considered as a highly aggressive ROS, since it is able to cause oxidative damage to almost any biological molecule, including proteins, DNA, nucleic acid, *etc*. [42]. One of the most applied *in vitro* methods for determination of the scavenging activity of a substance towards this species is based on the spectrophotometric measurement at 440 nm of the level of radical- induced oxidation of *N*,*N*-dimethyl-4-nitrosoaniline to *p*-nitrodimethylaniline, while a constant flux of HO^●^ is generated by a Fe^3+^/EDTA/H_2_O_2_/ascorbic acid system [43]. In contrast to the results obtained by the other two methods employed in the present study, the scavenging activities of compounds **3a**–**p** towards HO^●^ seem to be determined by the basic carbon backbone. As it can be seen, even compound **3a** (possessing no OH groups) showed satisfactory potential and this can be attributed to the ability of the carbon-carbon double bond to participate in reactions with HO^●^. Moreover, the introduction of a single or two, but “isolated”, phenolic groups, increases the activity of **3a**, and thus **3b**–**e** demonstrated nearly twofold lower EC_50_ values comparable with those of PCA and GA. However, as in case of O_2_^●▬^, the catechol fragment can be considered important, since the introduction of such a fragment additionally increased the activity of **3f** and **3g**. Similarly, the series of compounds follows the tendency for enhanced activity due to the presence of an extended π-conjugated system, and this can be rationalized by a comparison of the EC_50_ values of **3g** and **3i**, which resemble that of CA and PCA, respectively. Interestingly, the inclusion of a third OH group in ring B (pyrogallol fragment) in **3h** lowers its activity in comparison to **3g**, but the same behavior is also observed for the parent standards PCA and GA. Consequently, the introduction of a pyrogallol fragment is not necessary, since the activity of **3h** is slightly lower than that of **3b**–**e**, which possess reduced numbers of OH groups in ring B. The latter observation is noteworthy, since phenolic compounds with a single hydroxyl groups are less susceptible to oxidation reactions, and this advantage can be considered of immense importance from an industrial standpoint. Regarding compounds **3i**–**p**, each of them possessing the same catechol moiety in ring A and continuously increasing number of OH groups in ring B, it can be said that they obey the same tendency as **3a**–**g**, the latter underlining the importance of the catechol fragment to evoke antioxidant activity towards the HO^●^. 

### 2.3. Quantum Chemistry Computations 

**Figure 2:**
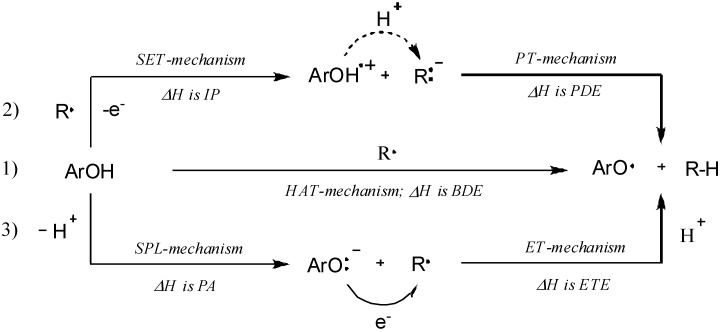
Probable reaction mechanisms of radical scavenging by phenolic compounds.

Three radical scavenging mechanisms stand out amongst the others proposed in the literature (see Figure 2). Two of them include hydrogen atom transfer (1)—the HAT-mechanism, and single-electron transfer (2)—the SET-PT-mechanism [44,45,46,47,48]. The HAT mechanism is based on homolytic dissociation of the O–H bond in phenolic compounds, and SET mechanism takes place in two steps—ionization, followed by a proton transfer. The third mechanism (SPL-ET) also proceeds in two steps [49,50], which include deprotonation at the first step followed by sequential electron transfer in the second. 

**Figure 3:**
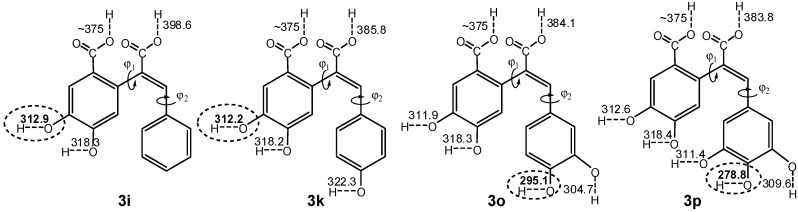
BDEs of compounds **3i**, **3k**, **3o** and **3p** for a homolytic dissociation of different bonds. Calculations are performed at IEF-PCM (U)B3LYP/6-31+G(d) level of theory.

In many cases two or more mechanisms can operate simultaneously during the antioxidant action, and, since the reaction path (1) is related to the bond dissociation energy—BDE; (2) to the ionization potential—IP, and proton dissociation enthalpy—PDE; and (3) to the proton affinity—PA, and electron transfer enthalpy—ETE, the calculation and comparison of these parameters can help distinguish the most probable reaction mechanism [51,52]. Therefore, in order to explain the apparent antioxidant activity and to assess the influence of the substitution pattern, the geometry and physical descriptors of compounds **3i**, **3k**, **3o** and **3p** were determined by means of DFT quantum chemical computations at the IEF-PCM (U)B3LYP/6-31+G(d) and (U)B3LYP/6-311++G(d,p) levels of theory. The computed data for all possible conformers of the corresponding molecule, radical, cation-radical and anion-radical species of the compounds of interest was found consistent with that experimentally obtained for a similar compound in a solid state [33], and showed that the carbon backbone is not planar and is composed of two independent fragments, e.g., protocatechuic and cinnamic acid fragments (ring A and ring B, respectively). The dihedral angle between the planes of these two parts (φ_1_, Figure 3) is in the range of 73–75°, the latter suggesting an absence of conjugation between them, and the aromatic ring in the cinnamic acid fragment is out of the plane with *ca.* 15–18° (φ_2_, Figure 3). Furthermore, it was found that the most stable species possess hydroxyl groups coplanar to the aromatic rings, orientated to each other in a way to form as much as possible intramolecular hydrogen bonds, and that the conversion of the molecule species into radicals, radical-cations and radical-anions does not lead to significant changes in the preferred conformations. 

The computed enthalpies for mechanisms (1)–(3), *i.e.*, BDE; IP; PDE; PA; ETE, are listed in Table 2. As it can be seen the BDE values are two- to threefold lower than the IP and PA, which suggests that the HAT mechanism is favored for the compounds under study and will always predominate over SET-PT and SPL-ET. As a rule of thumb, the HAT mechanism supposes a homolytic dissociation of the O–H bond, and more rarely, of a N–H and C–H bond. Therefore, when multiple groups are capable of participating in a reaction, the probability of a particular group to be “chosen” by the attacking radical can be assessed by a comparison of the corresponding BDEs. According to the values depicted in Figure 3, the BDEs of phenolic O–H groups, carboxylic O–H groups and C–H bonds are in the 278–323, 370–399, and 460–470 kJ·mol^−1^ range, respectively. Consequently, it can be concluded that the phenolic O–H groups are preferably attacked by selective radicals such as DPPH (BDE = 348 kJ·mol^−1^).

**Table 2:** IEF-PCM B3LYP/6-311++G** computed enthalpies (kJ·mol^−1^) of reaction mechanisms (1)–(3).

Compound	BDE ^a^	IP ^b^	PDE ^c^	PA ^d^	ETE ^e^
**3i**	324.4	826.2	169.0	759.2	684.8
**3k**	323.7	803.8	192.5	760.3	683.1
**3o**	306.4	796.1	189.2	749.3	676.6
**3p**	290.2	795.2	118.3	677.4	732.3

^a^ Bond Dissociation Energy; ^b^ Ionization Potential; ^c^ Proton Dissociation Enthalpy; ^d^ Proton Affinity; ^e^ Electron Transfer Enthalpy.

It is noteworthy that the influence of the substitution pattern of the target molecule is also of immense importance for the stability of the radicals produced, since the presence of groups capable of enhancing the resonance stabilization will facilitate the homolytic dissociation. Thus, due to the absence of phenolic groups in the cinnamic fragment in compound **3i**, participation in the reaction of the protocatechuic fragment, and more precisely the OH group *meta*-orientated toward the carboxylic function, will be preferred. This way, **3i** showed similar antioxidant activity compared to that of PCA. Similarly, the addition of a single OH group at 4 position in the cinnamic fragment in compound **3k** did not significantly affect the activity and this can be attributed to the absence of a neighboring group assuming additional stabilization. The importance of the latter can be rationalized by taking into account the BDEs of OH group at 4 position in the cinnamic fragment in compounds **3o** and **3p**, where the presence of one or two groups capable of stabilizing the resulting radical lowers the corresponding enthalpies, and hence facilitates the reaction to take place at this position. Based on the above arguments, it can be concluded that the major factor responsible for the apparent antioxidant activity of the compounds under study is the presence of at least one catechol fragment, regardless of which aromatic ring it is, and that in case where multiple centers are able to react, the presence of an extended π-conjugated system providing better delocalization, and thus stabilization of the radicals formed, will determine the preferred group to be attacked. This conclusion can also be supported by calculation of the spin densities, which show the probability a single electron to be found around given atom or atom groups. Moreover, since spin densities show the single electron distribution through the basic structure, they can serve as descriptors of the received radicals’ stability. 

To obtain these parameters for the radicals of compounds **3i**, **3k**, **3o** and **3p** we employed two approaches, namely Mulliken population analysis and Natural population analysis, and the results obtained are listed in Table 3. A short analysis of the data shows that the electron density in all cases is not concentrated only in the radical centre, but it is rather distributed either to the protocatechuic or to the cinnamic acid fragments, which in turn confirms the important role of these two fragments to evoke high antioxidant activities.

**Table 3:** Distribution of spin densities into radicals of compounds **3i**, **3k**, **3o** and **3p**.

Compound	Mulliken Spin Density			Natural Spin Density		
	Radical Centre	Protocatechuic Fragment	Cinnamic Fragment	Radical Centre	Protocatechuic Fragment	Cinnamic Fragment
**3i**	0.359	0.602	0.039	0.284	0.609	0.107
**3k**	0.337	0.608	0.055	0.269	0.666	0.065
**3o**	0.268	0.011	0.721	0.198	0.020	0.782
**3p**	0.287	0.038	0.675	0.218	0.021	0.761

## 3. Experimental Section 

### 3.1. General Remarks 

All chemicals used in this study were purchased from Sigma-Aldrich (FOT, Sofia, Bulgaria). The organic solvents were of analytical grade. Melting points were determined on a Kofler microscope Boetius PHMK 0.5 (VEB Kombinat Nagema, Radebeul, Germany). All reactions were monitored by means of thin layer chromatography (TLC) on pre-coated polyesters sheets POLIGRAM^®^ SIL G/UV254 (Merck, Darmstadt, Germany) and the spots were visualized with UV light. The column chromatography was performed on Horizon High Performance FLASH chromatography system (HPFC) with cartridges filled with Silica gel 60 (230–400 mesh, MACHEREY-NAGEL, Düren, Germany). The IR analyses were carried out on Cary 630 FTIR spectrophotometer (Agilent Technologies Inc., Santa Clara, CA, USA) equipped with diamond ATR-1 bounce and are reported in reciprocal centimeters. NMR spectra were recorded on а Bruker Avance II+ (600 MHz and 151 MHz for ^1^H and ^13^C, respectively) and Bruker Avance III HD (500 MHz and 126 MHz for ^1^H and ^13^C, respectively) using DMSO-*d*_6_ as а solvent and TMS as an internal standard. The chemicals shifts (δ) are given in ppm and *J* values are reported in Hz. Mass spectra were taken on Agilent 6890 series GC fitted with a HP-5MS capillary column (5% phenylmethylsiloxane, 30m × 250 μm × 0.25 μm, TEAM, Sofia, Bulgaria) with helium carrier gas (constant flow rate: 0.8 mL/min) coupled with an Agilent 5973 mass selective detector. Temperature programme: T(inlet): 250 °C, Oven: 150 °C (1 min), 150–300 °C (10 °C/min), 300 °C (5 min). Mass spectra: electron impact (EI+) mode (70 eV). In case of ^1^H-NMR, ^13^C-NMR and IR methods, the spectra were taken for the synthesized compounds, while their mass spectra were obtained after derivatization with diazomethane etherealsolution prior to analysis (see Section 3.2.2). The radical scavenging and antioxidant activity assays were performed on Thermo Evolution 60S UV/Vis spectrophotometer (ACM2, Sofia, Bulgaria).

### 3.2. Chemistry 

#### 3.2.1. General Procedure for One-Pot Synthesis of Polyhydroxy (*E*)-2-(1-Carboxy-2-phenylvinyl)-benzoic Acids **3a**–**p**

An equimolar mixture of homophtalic anhydride (**1a**) or 6,7-dimethoxyhomophthalic anhydride (**1b**), aldehyde **2a**–**h** and 4-dimethylaminopyridine (DMAP) in dry dichloromethane (10 mL) was stirred at room temperature while the consumption of the reagents was monitored by TLC. At the end of the reaction (*ca.* 10 min), boron tribromide (1.6 M solution in dichloromethane) was slowly added (one equivalent per heteroatom) and the reaction mixture was stirred additionally for 10–60 min. At the end of the reaction, the mixture was poured over ice, stirred for 10 min and acidified with conc. HCl (pH = 1). The aqueous layer was then saturated with NaCl and the products were extracted with ethyl acetate. The organic phase was washed with brine to a constant pH, dried over anhydrous Na_2_SO_4_ and the solvent was evaporated under reduced pressure. The residue was further purified by column chromatography (mobile phase: acetone/cyclohexane/formic acid—1/1/0.01) and the products **3a**–**p** were isolated after crystallization from appropriate mixture of solvents. Compounds **3a**, **3i**, **3k**, **3o** and **3p** were available from our previous works [31,33].

##### (*E*)-2-(1-Carboxy-2-(2-hydroxyphenyl)vinyl)benzoic Acid (**3b**)

Oil (0.32 g, 32%) after HPFC from the reaction of homophthalic anhydride (**1a**, 0.57 g, 3.52 mmol), 2-methoxybenzaldehyde (**2b**, 0.48 g, 3.52 mmol), 4-dimethylaminopyridine (0.43 g, 3.52 mmol) and boron tribromide (17.6 mL, 28.14 mmol) in dichloromethane (10 mL). Pale yellow crystals (0.18 g, 56%) after crystallization from 1:2 dichloromethane-acetone. M.p. = 180–182 °C; IR (ATR diamond, cm^−1^): ν = 3480 (OH), 3300–2250 (ОH, COOH), 1675 (C=O, COOH), 1630 (C=C); ^1^H-NMR (500 MHz, DMSO-*d*_6_): δ = 12.54 (bs, 2H, 2 × COOH), 9.96 (s, 1H, OH), 7.98 (d, 1H, *J* = 8.8 Hz, H8), 7.91 (s, 1H), 7.46–7.39 (m, 2H), 6.99 (dd, 2H, *J* = 8.1, 6.4 Hz), 6.83 (d, 1H, *J* = 8.1 Hz), 6.44–6.33 (m, 2H); ^13^C-NMR (126 MHz, DMSO-*d*_6_): δ = 168.1 (C1a), 167.7 (C7), 156.6 (C), 138.6 (C), 133.2 (C), 132.2 (=CH–), 131.7 (=CH–), 131.5 (C), 131.2 (=CH–), 130.3 (=CH–), 130.0 (=CH–), 129.6 (=CH–), 127.7 (=CH–), 121.7 (C), 118.5 (=CH–), 115.6 (=CH–); EIMS: *m/z* (%) = 326 (54) [M]^+^, 295 (100), 267 (43), 234 (44), 220 (41), 206 (26), 178 (66), 165 (61), 152 (42), 133 (35), 119 (21), 91 (21).

##### (*E*)-2-(1-Carboxy-2-(4-hydroxyphenyl)vinyl)benzoic Acid (**3c**)

Oil (0.89 g, 89%) after HPFC from the reaction of homophthalic anhydride (**1a**, 0.57 g, 3.52 mmol), 4-methoxybenzaldehyde (**2c**, 0.43 mL, 3.52 mmol), 4-dimethylaminopyridine (0.43 g, 3.52 mmol) and boron tribromide (17.6 mL, 28.14 mmol) in dichloromethane (10 mL). Pale yellow crystals (0.46 g, 52%) after crystallization from 1:5 dichloromethane-ethyl. M.p. = 166–167 °C; IR (ATR diamond, cm^−1^): ν = 3625 (OH), 3400–2200 (ОH, COOH), 1650 (C=O, COOH); ^1^H-NMR (600 MHz, DMSO-*d*_6_): δ = 12.48 (bs, 2H, 2 × COOH), 9.79 (s, 1H, OH), 8.01 (dd, 1H, *J* = 7.6, 1.1 Hz, H6), 7.54 (s, 1H, H8), 7.51 (td, 1H, *J* = 7.5, 1.5 Hz, H4), 7.47 (td, 1H, *J* = 7.5, 1.5 Hz, H5), 7.08 (dd, 1H, *J* = 7.6, 1.1 Hz, H3), 6.79 (d, 2H, *J* = 8.7 Hz, H2' and H6'), 6.55 (d, 2H, *J* = 8.7 Hz, H3' and H5'); ^13^C-NMR (151 MHz, DMSO-*d*_6_): δ = 168.2 (C1a), 167.6 (C7), 158.2 (C), 138.7 (C), 136.6 (C), 132.4 (=CH–), 132.0 (2 × =CH–), 131.3 (C), 131.2 (=CH–), 131.0 (C), 130.5 (=CH–), 127.8 (=CH–), 125.6 (=CH–), 115.3 (2 × =CH–); EIMS: *m/z* (%) = 326 (96) [M]^+^, 267 (63), 251 (33), 235 (100), 181 (20), 165 (45), 151 (48).

##### (*E*)-2-(1-Carboxy-2-(2,4-dihydroxyphenyl)vinyl)benzoic Acid (**3d**)

Oil (0.23 g, 23%) after HPFC from the reaction of homophthalic anhydride (**1a**, 0.54 g, 3.33 mmol), 2,4-dimethoxybenzaldehyde (**2d**, 0.55 g, 3.33 mmol), 4-dimethylaminopyridine (0.41 g, 3.33 mmol) and boron tribromide (18.7 mL, 29.97 mmol) in dichloromethane (10 mL). Pale yellow crystals (0.18 g, 80%) after crystallization from 1:2 dichloromethane-acetone. M.p. = 175–177 °C; IR (ATR diamond, cm^−1^): ν = 3500 (OH), 3300–2250 (ОH, COOH), 1675 (C=O, COOH), 1625 (C=C); ^1^H-NMR (500 MHz, DMSO-*d*_6_): δ = 12.35 (bs, 2H, 2 × COOH), 9.87 (s, 1H, OH), 9.58 (s, 1H, OH), 7.96 (dd, 1H, *J* = 7.7, 1.4 Hz, H6), 7.88 (s, 1H, H8), 7.46 (td, 1H, *J* = 7.5, 1.5 Hz, H4), 7.41 (td, 1H, *J* = 7.5, 1.5 Hz, H5), 7.03 (dd, 1H, *J* = 7.5, 1.2 Hz, H3), 6.29 (d, 1H, *J* = 2.4 Hz, H3'), 6.14 (d, 1H, *J* = 8.7 Hz, H6'), 5.81 (dd, 1H, *J* = 8.7, 2.4 Hz, H5'); ^13^C-NMR (126 MHz, DMSO-*d*_6_): δ = 168.4 (C1a), 167.8 (C7), 159.4 (C), 158.3 (C), 139.2 (C), 132.2 (=CH–), 131.7 (=CH–), 131.5 (C), 131.3 (=CH–), 130.5 (=CH–), 130.3 (=CH–), 129.2 (C), 127.4 (=CH–), 113.0 (C), 106.7 (=CH–), 102.1 (=CH–); EIMS: *m/z* (%) = 356 (100) [M]^+^, 325 (45), 297 (29), 265 (21), 250 (30), 235 (26), 181 (36), 165 (40), 152 (34), 133 (20).

##### (*E*)-2-(1-Carboxy-2-(2,5-dihydroxyphenyl)vinyl)benzoic Acid (**3e**)

Orange solid foam (0.14 g, 14%) after HPFC from the reaction of homophthalic anhydride (**1a**, 0.54 g, 3.33 mmol), 2,5-dimethoxybenzaldehyde (**2e**, 0.55 g, 3.33 mmol), 4-dimethylaminopyridine (0.41 g, 3.33 mmol) and boron tribromide (18.7 mL, 29.97 mmol) in dichloromethane (10 mL). M.p. = 108–110 °C; IR (ATR diamond, cm^−1^): ν = 3480 (OH), 3300–2250 (ОH, COOH), 1675 (C=O, COOH), 1630 (C=C); ^1^H-NMR (500 MHz, DMSO-*d*_6_): δ = 12.50 (s, 2H, 2 × COOH), 9.23 (s, 1H, OH), 8.44 (s, 1H, OH), 7.99 (dd, 1H, *J* = 7.4, 1.6 Hz, H6), 7.85 (s, 1H, H8), 7.44 (td, 2H, *J* = 7.4, 1.6 Hz, H4 and H5), 6.98 (dd, 1H, *J* = 7.4, 1.6 Hz, H3), 6.63 (d, 1H, *J* = 8.7 Hz, H3'), 6.46 (dd, 1H, *J* = 8.7, 2.9 Hz, H4'), 5.83 (d, 1H, *J* = 2.9 Hz, H6'); ^13^C-NMR (126 MHz, DMSO-*d*_6_): δ = 168.2 (C1a), 167.7 (C7), 149.4 (C), 149.0 (C), 138.6 (C), 132.9 (C), 132.2 (=CH–), 131.9 (=CH–), 131.4 (C), 131.2 (=CH–), 130.3 (=CH–), 127.6 (=CH–), 122.1 (C), 117.3 (=CH–), 116.0 (=CH–), 115.7 (=CH–); EIMS: *m/z* (%) = 356 (79) [M]^+^, 325 (100), 297 (24), 265 (22), 250 (47), 207 (52), 179 (22), 165 (40), 152 (45), 139 (24), 59 (25). 

##### (*E*)-2-(1-Carboxy-2-(2,3-dihydroxyphenyl)vinyl)benzoic Acid (**3f**)

Brown solid foam (0.20 g, 20%) after HPFC from the reaction of homophthalic anhydride (**1a**, 0.54 g, 3.33 mmol), 2,3-dimethoxybenzaldehyde (**2f**, 0.55 g, 3.33 mmol), 4-dimethylaminopyridine (0.41 g, 3.33 mmol) and boron tribromide (18.7 mL, 29.97 mmol) in dichloromethane (10 mL). M.p. = 137–138 °C; IR (ATR diamond, cm^−1^): ν = 3630 (OH), 3500–2200 (ОH, COOH), 1670 (C=O, COOH); ^1^H-NMR (500 MHz, DMSO-*d*_6_): δ = 12.52 (bs, 2H, 2 × COOH), 9.47 (s, 1H, OH), 8.85 (s, 1H, OH), 7.97 (dd, 1H, *J* = 6.8, 2.3 Hz, H6), 7.93 (s, 1H, H8), 7.46–7.38 (m, 2H, H4 and H5), 6.99 (dd, 1H, *J* = 6.7 Hz, H3), 6.61 (dd, 1H, *J* = 7.8, 1.4 Hz, H6'), 6.22 (t, 1H, *J* = 7.9 Hz, H5'), 5.84 (dd, 1H, *J* = 8.0, 1.1 Hz, H4'); ^13^C-NMR (126 MHz, DMSO-*d*_6_): δ = 168.1 (C1a), 167.7 (C7), 145.2 (C), 145.1 (C), 133.0 (C), 132.1 (=CH–), 132.0 (=CH–), 131.4 (C), 131.3 (=CH–), 130.2 (=CH–), 127.6 (=CH–), 122.5 (C), 120.0 (=CH–), 118.2 (=CH–), 115.3 (=CH–); EIMS: *m/z* (%) = 356 (23) [M]^+^, 325 (100), 265 (25), 165 (21). 

##### (*E*)-2-(1-Carboxy-2-(3,4-dihydroxyphenyl)vinyl)benzoic Acid (**3g**)

Oil (0.51 g, 51%) after HPFC from the reaction of homophthalic anhydride (**1a**, 0.54 g, 3.33 mmol), 3,4-dimethoxybenzaldehyde (**2g**, 0.55 g, 3.33 mmol), 4-dimethylaminopyridine (0.41 g, 3.33 mmol) and boron tribromide (18.7 mL, 29.97 mmol) in dichloromethane (10 mL). Beige crystals (0.35 g, 68%) after crystallization from 1:2 petroleum ether-ethyl acetate. M.p. = 192–194 °C; IR (ATR diamond, cm^−1^): ν = 3575 (OH), 3500–2250 (ОH, COOH), 1670 (C=O, COOH), 1610 (C=C); ^1^H-NMR (600 MHz, DMSO-*d*_6_): δ = 12.45 (bs, 2H, 2 × COOH), 9.34 (s, 1H, OH), 8.81 (s, 1H, OH), 8.01 (dd, 1H, *J* = 7.5, 1.3 Hz, H6), 7.51 (td, 1H, *J* = 7.5, 1.3 Hz, H4), 7.46 (td, 1H, *J* = 7.5, 1.3 Hz, H5), 7.45 (s, 1H, H8), 7.07 (dd, 1H, *J* = 7.5, 1.3 Hz, H3), 6.53 (d, 1H, *J =* 8.8 Hz, H5'), 6.33 (td, 2H, *J* = 4.3, 1.9 Hz, H2' and H6'); ^13^C-NMR (151 MHz, DMSO-*d*_6_): δ = 168.2 (C1a), 167.6 (C7), 146.7 (C), 144.8 (C), 138.8 (C), 137.0 (C), 132.4 (=CH–), 131.3 (=CH–) 131.2 (C), 130.9 (C), 130.5 (=CH–), 127.8 (=CH–), 126.1 (=CH–), 122.9 (=CH–), 117.5 (=CH–), 115.3 (=CH–); EIMS: *m/z* (%) = 356 (99) [M]^+^, 297 (30), 265 (100), 238 (28), 221 (20), 181 (37), 165 (35), 152 (35), 139 (20).

##### (*E*)-2-(1-Carboxy-2-(3,4,5-trihydroxyphenyl)vinyl)benzoic Acid (**3h**)

Oil (0.74 g, 74%) after HPFC from the reaction of homophthalic anhydride (**1a**, 0.51 g, 3.16 mmol), 3,4,5-trimethoxybenzaldehyde (**2h**, 0.62 g, 3.16 mmol), 4-dimethylaminopyridine (0.39 g, 3.16 mmol) and boron tribromide (19.8 mL, 31.62 mmol) in dichloromethane (10 mL). Yellow crystals (0.27 g, 36%) after crystallization from 1:5 dichloromethane-ethyl acetate. M.p. = 158–160 °C; IR (ATR diamond, cm^−1^): ν = 3640 (ОH), 3580–2180 (ОH, COOH), 1665 (C=O, COOH), 1630 (C=C); ^1^H-NMR (600 MHz, DMSO*-d*_6_): δ = 12.45 (s, 2H, 2 × COOH), 8.79 (s, 2H, 2 × OH), 8.54 (s, 1H, OH), 8.00 (dd, 1H, *J* = 7.6, 1.3 Hz, H6), 7.49 (td, 1H, *J* = 7.6, 1.3 Hz, H4), 7.45 (td, 1H, *J* = 7.6, 1.3 Hz, H5), 7.35 (s, 1H, H8), 7.05 (dd, 1H, *J* = 7.6, 1.3 Hz, H3), 5.94 (s, 2H, H2' and H6'); ^13^C-NMR (151 MHz, DMSO*-d*_6_): δ = 168.3 (C1а), 167.7 (C7), 145.5 (3 × C), 138.7 (C), 137.3 (C), 134.8 (=CH–), 132.3 (C), 131.3 (=CH–), 130.5 (=CH–), 127.7 (C), 125.0 (=CH–), 109.9 (3 × =CH–); EIMS: *m/z* (%) = 386 (100) [M]^+^, 311 (43), 295 (35), 183 (20), 139 (24).

##### (*E*)-2-(1-Carboxy-2-(2-hydroxyphenyl)vinyl)-4,5-dihydroxybenzoic Acid (**3j**)

Оil (0.23 g, 23%) after HPFC from the reaction of 6,7-dimethoxyhomophthalic anhydride (**1b**, 0.70 g, 3.16 mmol), 2-methoxybenzaldehyde (**2b**, 0.43 g, 3.16 mmol), 4-dimethylaminopyridine (0.39 g, 3.16 mmol) and boron tribromide (19.8 mL, 31.62 mmol) in dichloromethane (10 mL). Yellow crystals (0.18 g, 77%) after crystallization from 1:2 dichloromethane-acetone. M.p. = 179–180 °C; IR (ATR diamond, cm^−1^): ν = 3670–2160 (ОH, COOH), 1656 (C=O, COOH), 1620 (C=C); ^1^H-NMR (600 MHz, DMSO-*d*_6_): δ = 11.94 (bs, 2H, 2 × COOH), 9.69 (s, 1H, OH), 9.37 (s, 1H, OH), 9.08 (s, 1H, OH), 7.80 (s, 1H, H8), 7.43 (s, 1H, H6), 7.00 (t, 1H, *J* = 7.5 Hz, H4'), 6.82 (d, 1H, *J* = 7.7 Hz, H6'), 6.53 (d, 1H, *J* = 7.7 Hz, H3'), 6.46 (t, 1H, *J* = 7.5 Hz, H5'), 6.33 (s, 1H, H3); ^13^C-NMR (151 MHz, DMSO-*d*_6_): δ = 168.3 (C1a), 167.1 (C7), 156.3 (C), 149.0 (C), 144.1 (C), 133.5 (C), 131.2 (=CH–), 130.4 (C), 129.5 (2 × =CH–), 122.2 (C), 121.7 (C), 118.6 (=CH–), 118.0 (=CH–), 117.8 (=CH–), 115.5 (=CH–); EIMS: *m/z* (%) = 386 (100) [M]^+^, 355 (43), 327 (80), 295 (63), 281 (31), 265 (38), 251 (24), 237 (20), 223 (28), 205 (29), 193 (55), 181 (44), 165 (51), 152 (46), 139 (38), 119 (21), 91 (33), 77 (30), 59 (58), 51 (25).

##### (*E*)-2-(1-Carboxy-2-(2,4-dihydroxyphenyl)vinyl)-4,5-dihydroxybenzoic Acid (**3l**)

Оil (0.16 g, 16%) after HPFC from the reaction of 6,7-dimethoxyhomophthalic anhydride (**1b**, 0.67 g, 3.01 mmol), 2,4-dimethoxybenzaldehyde (**2d**, 0.50 g, 3.01 mmol), 4-dimethylaminopyridine (0.37 g, 3.01 mmol) and boron tribromide (20.7 mL, 33.11 mmol) in dichloromethane (10 mL). Pale yellow crystals (0.13 g, 81%) after crystallization from 1:2 dichloromethane-acetone. M.p. = 182–183 °C; IR (ATR diamond, cm^−1^): ν = 3640 (OH), 3560 (OH), 3500–2160 (ОH, COOH), 1670 (C=O, COOH), 1615 (C=C); ^1^H-NMR (600 MHz, DMSO-*d*_6_): δ = 11.98 (bs, 2H, 2 × COOH), 9.81 (s, 1H, OH), 9.56 (s, 2H, 2 × OH), 9.26 (bs, 1H, OH), 7.74 (s, 1H, H8), 7.41 (s, 1H, H6), 6.34 (s, 1H, H3), 6.30–6.28 (m, 2H, H3' and H6'), 5.89 (dd, 1H, *J* = 8.7, 2.3 Hz, H5'); ^13^C-NMR (151 MHz, DMSO-*d*_6_): δ = 168.6 (C1a), 167.1 (C7), 159.0 (C), 157.8 (C), 148.9 (C), 143.8 (C), 131.7 (C), 130.4 (C), 130.2 (=CH–), 129.7 (C), 121.7 (C), 117.9 (=CH–), 117.8 (=CH–), 113.4 (=CH–), 106.6 (=CH–), 102.1 (=CH–); EIMS: *m/z* (%) = 416 (100) [M]^+^, 357 (38), 325 (24), 181 (81), 149 (25), 59 (39).

##### (*E*)-2-(1-Carboxy-2-(2,5-dihydroxyphenyl)vinyl)-4,5-dihydroxybenzoic Acid (**3m**)

Oil (0.21 g, 21%) after HPFC from the reaction of 6,7-dimethoxyhomophthalic anhydride (**1b**, 0.67 g, 3.01 mmol), 2,5-dimethoxybenzaldehyde (**2e**, 0.50 g, 3.01 mmol), 4-dimethylaminopyridine (0.37 g, 3.01 mmol) and boron tribromide (20.7 mL, 33.11 mmol) in dichloromethane (10 mL). Pale yellow crystals (0.15 g, 71%) after crystallization from 1:2 dichloromethane-acetone. M.p. = 173–174 °C; IR (ATR diamond, cm^−1^): ν = 3600 (OH), 3520–2160 (ОH, COOH), 1675 (C=O, COOH); ^1^H-NMR (600 MHz, DMSO-*d*_6_): δ = 12.09 (s, 2H, 2 × COOH), 9.58 (s, 1H, OH), 9.28 (s, 1H, OH), 9.18 (s, 1H, OH), 8.52 (s, 1H, OH), 7.73 (s, 1H, H8), 7.43 (s, 1H, H6), 6.62 (d, 1H, *J* = 8.7 Hz, H3'), 6.47 (dd, 1H, *J* = 8.7, 3.0 Hz, H4'), 6.31 (s, 1H, H3), 5.97 (d, 1H, *J* = 3.0 Hz, H6'); ^13^C-NMR (151 MHz, DMSO-*d*_6_): δ = 168.7 (C1a), 167.3 (C7), 149.4 (C), 149.1 (C), 149.0 (C), 144.2 (C), 133.2 (C), 131.2 (C), 130.6 (C), 122.5 (=CH–), 121.5 (C), 118.1 (=CH–), 117.8 (=CH–), 117.1 (=CH–), 115.9 (=CH–), 115.8 (=CH–); EIMS: *m/z* (%) = 416 (100) [M]^+^, 385 (71), 357 (41), 325 (55), 310 (28), 295 (21), 283 (24), 267 (40), 253 (21), 225 (25), 209 (23), 193 (26), 181 (38), 165 (21), 149 (28), 139 (24), 59 (70).

##### (*E*)-2-(1-Carboxy-2-(2,3-dihydroxyphenyl)vinyl)-4,5-dihydroxybenzoic Acid (**3n**)

Oil (0.37 g, 37%) after HPFC from the reaction of 6,7-dimethoxyhomophthalic anhydride (**1b**, 0.67 g, 3.01 mmol), 2,3-dimethoxybenzaldehyde (**2f**, 0.50 g, 3.01 mmol), 4-dimethylaminopyridine (0.37 g, 3.01 mmol) and boron tribromide (20.7 mL, 33.11 mmol) in dichloromethane (10 mL). Beige crystals (0.20 g, 54%) after crystallization from 1:2 dichloromethane-acetone. M.p. = 180–182 °C; IR (ATR diamond, cm^−1^): ν = 3630 (OH) 3550–2160 (ОH, COOH), 1670 (C=O, COOH); ^1^H-NMR (600 MHz, DMSO-*d*_6_): δ = 12.11 (bs, 2H, 2 × COOH), 9.57 (s, 1H, OH), 9.45 (s, 1H, OH), 9.27 (s, 1H, OH), 8.79 (s, 1H, OH), 7.80 (s, 1H, H8), 7.41 (s, 1H, H6), 6.61 (dd, 1H, *J* = 7.9, 1.2 Hz, H6'), 6.31 (s, 1H, H3), 6.29 (t, 1H, *J* = 7.9 Hz, H5'), 5.98 (dd, 1H, *J* = 7.9, 1.2 Hz, H4'); ^13^C-NMR (151 MHz, DMSO-*d*_6_): δ = 168.6 (C1а), 167.3 (C7), 149.1 (C), 145.2 (C), 145.0 (C), 144.2 (C), 133.4 (C), 131.4 (C), 130.8 (C), 122.8 (=CH–), 121.5 (C), 120.1 (=CH–), 118.3 (=CH–), 118.0 (=CH–), 117.9 (=CH–), 115.1 (=CH–); EIMS: *m/z* (%) = 416 (56) [M]^+^, 385 (65), 357 (23), 325 (100), 298 (20), 267 (27), 193 (31), 181 (24), 139 (20), 59 (50).

#### 3.2.2. General Procedure for Derivatization of Polyhydroxy (*E*)-2-(1-Carboxy-2-phenylvinyl)benzoic Acids **3a**–**p**

To each compound (1 mg), ethereal diazomethane solution was slowly added until complete dissolution. The end of the reaction was determined by means of TLC. After that, the solvent was evaporated under reduced pressure, the residue was dissolved in dichloromethane and analyzed as described in Section 3.1.

### 3.3. In Vitro Antioxidant Capacity Assays

#### 3.3.1. 1,1-Diphenyl-2-picrylhydrazyl Radical Scavenging Assay (DPPH^●^) 

A methanolic solution (100 μM) of the DPPH^●^ radical was prepared daily and protected from light. Absorbance was recorded to check the stability of the radical throughout the time of analysis. Five different concentrations of DPPH^●^ radical (in the range 100–10 μM) were also prepared every day and a linear relationship between radical concentration and absorbance was established. The effect of tested compounds on the DPPH^●^ absorbance was estimated by using a modified procedure described by Brand-Williams [53]. Different analyte concentrations dissolved in methanol (0.5 mL) were added to DPPH^●^ methanolic solution (0.5 mL). Absorbance at 518 nm was recorded at different time intervals until the reaction reached equilibrium (plateau). The initial absorbance was close to 0.600 in all cases. The blank reference cuvette contained methanol. All measurements were performed in triplicate. Five different concentrations of each phenolic compound have been assayed in order to check the linearity of response and to establish the antioxidant activity values in the adequate linear range. The end of the reaction for each compound is simultaneously dependent on its reactivity and concentration, therefore this issue was addressed by introduction of an additional parameter—TEC_50_—the time required to reach equilibrium at a concentration of compound at EC_50_.

#### 3.3.2. Superoxide Anion Radical Scavenging Assay (O_2_^●▬^)

A NADH/PMS/NBT system was used to determine the superoxide anion scavenging activities of the compounds as described by Suzumura *et al*. [54]. Briefly, generation of superoxide anion was measured in a reaction mixture containing 600 μM NADH, 150 μM NBT and 30 μM PMS in phosphate buffered saline (PBS) at pH = 7.4. The reduction of NBT was followed by measuring the change in absorbance at 560 nm for 10 min, a period in which the absorbance increases linearly. Test compounds with different concentrations were prepared in methanol and added to the reaction mixture, whereafter their activity was determined in comparison with the control sample which did not contain the test compound.

#### 3.3.3. Hydroxyl Radical Scavenging Assay (HO^●^)

Hydroxyl radical scavenging activity was measured as described by Kunchandy and Rao [55], with slight modifications. Hydroxyl radical generated through Fenton reaction, can bleach *p*-nitroso-dimethyl aniline (*p*-NDA) specifically. Scavenging activity was measured by the extent of inhibition of bleaching of *p*-NDA in presence of the test compound. To a reaction mixture containing FeCl_3_/EDTA (0.1/0.1 mM), H_2_О_2_ (2 mM), *p*-NDA (0.01 mM), different concentrations of test compound in phosphate buffer pH = 7.4 (20 mM), was added ascorbic acid (0.1 mM) to give final volume of 1.5 mL. Absorbance was measured at 440 nm. Percentage scavenging was calculated from the control where not test compound was present.

### 3.4. Statistical Analysis 

All analyses were performed in triplicate. Results were expressed as mean EC_50_ ± SD (μM). Statistical analyses of the data were performed with one-way analysis of variance (ANOVA) from summary data. The averages were statistically compared and this was taken into account in the discussion of the antioxidant capacity. A level of confidence higher than 95% (*p* < 0.05) was considered as statistically significant.

### 3.5. Theoretical Approach

All computations were performed by means of the Gaussian-09 program package [56]. The geometries of all possible conformers of the studied species were fully optimized by application of B3LYP hybrid functional [57] on 6-31+G(d) and 6-31++G(d,p) basis sets. The geometries of open shell species (radicals and cation-radicals) were optimized on the same theory levels by unrestricted wave functions (UB3LYP). The stationary points found on the potential energy hypersurfaces for each structure, were characterized using the standard harmonic vibrational analysis. The absence of imaginary frequencies confirmed that the stationary points corresponded to a minimum on the potential hypersurfaces. Since the free radical-scavenging activity strongly depends on medium polarity, solvent effect was taken into account in this study. For this aim, Integral Equation Formalism version of Tomasi’s Polarizable Continuum Model (IEF-PCM) [58] was used for all geometry optimizations and calculation of descriptors. We choose water as solvent to represent real biological systems. All enthalpies were calculated for 298 K. The values of BDE, IP, PDE, PA, ETE were determined from total enthalpies of the individual species, as follows:

BDE = H(ArO●) + H(H●) − H(ArOH)(1)

IP = H(ArOH+●) + H(e−) − H(ArOH)(2)

PDE = H(ArO●) + H(H+) − H(ArOH+●)(3)

PA = H(ArO−) + H(H+) − H(ArOH)(4)

ETE = H(ArO●) + H(e−) − H(ArO−)(5)

Besides enthalpies of ArO^●^, ArOH^+●^, ArO^−^ the enthalpies of hydrogen radical H^●^ and proton were also calculated at the same level of theory. The value of enthalpy of hydrated electron was taken from the literature [59]. The spin densities were obtained from Mulliken and from Natural Population Analysis. 

## 4. Conclusions 

In this work, we have evaluated the antioxidant capacity of a series of hybrid molecules, composed of natural phenolic acids such as benzoic, protocatechuic, cinnamic, *p*-coumaric, caffeic, or gallic acids, against DPPH^●^, HO^●^ and O_2_^●▬^ radicals. The results obtained showed that most of the tested compounds are highly effective in micromolar concentrations, possessing higher activities than well-known natural antioxidants. Based on the performed SAR analysis and DFT quantum chemical computations, it was deduced that, in comparison with their number, the distribution of the OH groups in the basic carbon backbone contributes in a greater extent to the radical scavenging activity, which is consistent with previously described relationships [60]. It was also shown that the major factor responsible for the apparent activity of the compounds under study is the presence of at least one catechol fragment, regardless in which aromatic ring it is on, and that in case of multiple centers are able to react, the presence of an extended π-conjugated system providing better delocalization, and thus stabilization of the radicals formed, will determine the preferred group to be attacked. In sum, the catechol fragment was shown to be of higher impact toward the scavenging activity against OH^●^ radical, whereas pyrogallol fragment – toward O_2_^●▬^. On the other hand, the absence of correlations between the results obtained by different methods suggests that different mechanism of action can operate, and that the lack of activity against DPPH^●^ does not mean that the compounds do not possess antioxidant activities and *vice versa*. Based on the above reasoning, it can be concluded that the synthesis of hybrid antioxidants is credible, and that additional work toward the evaluation of their potential as drug-candidates for preventing oxidative stress associated diseases is worth to be performed. 

## Supplementary Materials

Supplementary materials can be accessed at: http://www.mdpi.com/1420-3049/20/02/2555/s1.
