# Supplementary material for: Synthesis and Antioxidant Activity of Polyhydroxylated trans-Restricted 2-Arylcinnamic Acids
Source: Molecules. 2015 Feb 2;20(2):2555–75. doi: 10.3390/molecules20022555 (PMC6272747; doi:10.3390/molecules20022555)
Supplement: Supplementary file 1 [file molecules-20-02555-s001.pdf]

Supporting Information

Table S1. *p*-Values of the relative activity ($EC_{50} \pm SD$) against DPPH$^{\bullet}$ radical of all possible pairs of compounds.

Compound	3a	3b	3c	3d	3e	3f	3g	3h	3i	3j	3k	3l	3m	3n	3o	3p	Trolox	CA	PCA	GA	CA:PCA
**3a**		–	–	–	–	–	–	–	–	–	–	–	–	–	–	–	–	–	–	–	–
**3b**			–	–	–	–	–	–	–	–	–	–	–	–	–	–	–	–	–	–	–
**3c**				–	– *	–	–	–	–	–	–	–	–	–	–	–	–	–	–	–	–
**3d**					+ *	+	+	+	+	+	+	+	+	+	+	+	+	+	+	+	+
**3e**						0.272	0.136	+	+	+	0.056	+	+	+	+	+	+	+	+	+	+
**3f**							0.778	+	+	+	+	0.100	+	+	+	+	+	+	+	0.071	+
**3g**								+	+	+	+	0.106	+	+	+	+	+	+	+	0.071	+
**3h**									+	+	+	+	+	+	+	+	+	+	+	+	+
**3i**										0.162	0.266	+	+	+	+	+	+	+	+	+	+
**3j**											+	+	+	+	+	+	+	+	+	+	+
**3k**												+	+	+	+	+	+	+	+	+	+
**3l**													+	+	+	+	+	+	+	0.720	+
**3m**														+	+	+	+	+	+	+	+
**3n**															+	+	+	+	+	+	+
**3o**																+	+	+	+	+	+
**3p**																	+	+	+	+	+
Trolox																		0.258	+	+	+
CA																			+	+	+
PCA																				+	+
GA																					+
CA:PCA																					

* "–" refers to "not determined" due to lack of activity and "+" refers to a level of confidence higher than 95% ($p < 0.05$).

Table S2. *p*-Values of the relative activity ($EC_{50} \pm SD$) against $O_2^{\bullet-}$ anion radical of all possible pairs of compounds.

Compound	3a	3b	3c	3d	3e	3f	3g	3h	3i	3j	3k	3l	3m	3n	3o	3p	Trolox	CA	PCA	GA	CA:PCA
**3a**		–	–	–	–	–	–	–	–	–	–	–	–	–	–	–	–	–	–	–	–
**3b**			–	–	–	–	–	–	–	–	–	–	–	–	–	–	–	–	–	–	–
**3c**				–	–	–	–	–	–	–	–	–	–	–	–	–	–	–	–	–	–
**3d**					–	–	–	–	–	–	–	–	–	–	–	–	–	–	–	–	–
**3e**						–	– *	–	–	–	–	–	–	–	–	–	–	–	–	–	–
**3f**							+ *	+	+	+	+	+	–	+	+	+	–	+	+	+	+
**3g**								+	+	+	+	+	–	+	+	+	–	0.077	+	+	+
**3h**									+	+	+	+	–	+	+	0.820	–	+	+	+	+
**3i**										0.235	0.368	+	–	+	+	+	–	+	0.576	+	+
**3j**											0.157	+	–	+	+	+	–	+	0.197	+	+
**3k**												+	–	+	+	+	–	+	0.615	+	+
**3l**													–	+	+	+	–	+	+	+	+
**3m**														–	–	–	–	–	–	–	–
**3n**															+	+	–	0.163	+	+	0.171
**3o**																+	–	+	+	+	+
**3p**																	–	+	+	+	+
Trolox																		–	–	–	–
CA																			+	+	0.063
PCA																				+	+
GA																					+
CA:PCA																					

* "–" refers to "not determined" due to lack of activity and "+" refers to a level of confidence higher than 95% ($p < 0.05$).

Table S3. *p*-Values of the relative activity ($EC_{50} \pm SD$) against $HO^{\bullet}$ radical of all possible pairs of compounds.

Compound	3a	3b	3c	3d	3e	3f	3g	3h	3i	3j	3k	3l	3m	3n	3o	3p	Trolox	CA	PCA	GA	CA:PCA
**3a**		+ *	+	+	+	+	+	+	+	+	+	+	+	+	+	+	+	+	+	+	+
**3b**			0.964	0.303	0.310	0.114	+	+	0.187	+	+	+	+	+	+	+	0.079	+	0.161	0.240	0.875
**3c**				0.180	+	+	+	+	+	+	+	+	+	+	+	+	+	+	+	+	0.871
**3d**					0.749	0.290	+	+	0.733	+	+	+	+	+	+	+	0.190	+	0.558	+	0.191
**3e**						0.162	+	+	0.454	+	+	+	+	+	+	+	+	+	+	+	0.168
**3f**							+	0.363	0.296	+	+	+	+	+	+	+	0.948	+	0.341	+	0.072
**3g**								+	+	+	+	+	0.433	+	+	0.210	+	+	+	+	+
**3h**									+	+	+	+	+	+	+	+	0.126	+	+	+	+
**3i**										+	+	+	+	+	+	+	0.125	+	0.590	+	0.093
**3j**											+	+	+	+	+	+	+	+	+	+	+
**3k**												+	+	+	+	+	+	+	+	+	+
**3l**													+	+	+	+	+	+	+	+	+
**3m**														+	+	0.759	+	+	+	+	+
**3n**															+	+	+	+	+	+	+
**3o**																+	+	+	+	+	+
**3p**																	+	+	+	+	+
Trolox																		+	0.153	+	+
CA																			+	+	+
PCA																				+	+
GA																					0.231
CA:PCA																					

* "+" refers to a level of confidence higher than 95% ($p < 0.05$).
